# Untargeted metabolomics reveals plasma metabolites predictive of ectopic fat in pancreas and liver as assessed by magnetic resonance imaging: the TOFI_Asia study

**DOI:** 10.1038/s41366-021-00854-x

**Published:** 2021-05-16

**Authors:** Zhanxuan E. Wu, Karl Fraser, Marlena C. Kruger, Ivana R. Sequeira, Wilson Yip, Louise W. Lu, Lindsay D. Plank, Rinki Murphy, Garth J. S. Cooper, Jean-Charles Martin, Kieren G. Hollingsworth, Sally D. Poppitt

**Affiliations:** 1 Food Nutrition & Health, Food and Bio-based Products, AgResearch Limited, Palmerston North, New Zealand; 2 School of Health Sciences, Massey University, Palmerston North, New Zealand; 3 High-Value Nutrition National Science Challenge, Auckland, New Zealand; 4 Riddet Institute, Massey University, Palmerston North, New Zealand; 5 Human Nutrition Unit, School of Biological Sciences, University of Auckland, Auckland, New Zealand; 6 Department of Surgery, University of Auckland, Auckland, New Zealand; 7 Department of Medicine, University of Auckland, Auckland, New Zealand; 8 School of Biological Sciences University of Auckland, Auckland, New Zealand; 9 Centre for Advanced Discovery and Experimental Therapeutics, School of Medical Sciences, University of Manchester, Manchester, UK; 10 Aix-Marseille University, INSERM, INRAe, C2VN, BioMeT, Marseille, France; 11 Translational and Clinical Research Institute, Faculty of Medical Sciences, Newcastle University, Newcastle upon Tyne, UK

**Keywords:** Obesity, Lipidomics, Risk factors

## Abstract

**Background:**

Excess visceral obesity and ectopic organ fat is associated with increased risk of cardiometabolic disease. However, circulating markers for early detection of ectopic fat, particularly pancreas and liver, are lacking.

**Methods:**

Lipid storage in pancreas, liver, abdominal subcutaneous adipose tissue (SAT) and visceral adipose tissue (VAT) from 68 healthy or pre-diabetic Caucasian and Chinese women enroled in the TOFI_Asia study was assessed by magnetic resonance imaging/spectroscopy (MRI/S). Plasma metabolites were measured with untargeted liquid chromatography–mass spectroscopy (LC–MS). Multivariate partial least squares (PLS) regression identified metabolites predictive of VAT/SAT and ectopic fat; univariate linear regression adjusting for potential covariates identified individual metabolites associated with VAT/SAT and ectopic fat; linear regression adjusted for ethnicity identified clinical and anthropometric correlates for each fat depot.

**Results:**

PLS identified 56, 64 and 31 metabolites which jointly predicted pancreatic fat (R2Y = 0.81, Q2 = 0.69), liver fat (RY2 = 0.8, Q2 = 0.66) and VAT/SAT ((R2Y = 0.7, Q2 = 0.62)) respectively. Among the PLS-identified metabolites, none of them remained significantly associated with pancreatic fat after adjusting for all covariates. Dihydrosphingomyelin (dhSM(d36:0)), 3 phosphatidylethanolamines, 5 diacylglycerols (DG) and 40 triacylglycerols (TG) were associated with liver fat independent of covariates. Three DGs and 12 TGs were associated with VAT/SAT independent of covariates. Notably, comparison with clinical correlates showed better predictivity of ectopic fat by these PLS-identified plasma metabolite markers.

**Conclusions:**

Untargeted metabolomics identified candidate markers of visceral and ectopic fat that improved fat level prediction over clinical markers. Several plasma metabolites were associated with level of liver fat and VAT/SAT ratio independent of age, total and visceral adiposity, whereas pancreatic fat deposition was only associated with increased sulfolithocholic acid independent of adiposity-related parameters, but not age.

## Introduction

Although obesity has been long recognised as a risk factor for cardiometabolic disease and subsequent complications [1], individuals within each body mass index (BMI) category show considerable heterogeneity in their cardiometabolic manifestations and clinical risk profiles [2]. Notably specific populations, e.g., South and East Asians, develop type 2 diabetes (T2D) at lower BMI and younger age [3], with risk of T2D increased by even modest weight gain compared to more resilient populations such as Caucasians [4]. One factor purported to drive these risks among individuals with comparable BMI is deposition of visceral and non-adipose ectopic organ fat [5], likely a contributor to ethnicity differences in progression to T2D [6]. Asians have been observed to have greater propensity for abdominal and ectopic fat deposition, compared with other ethnicities [7–9]. Visceral and ectopic fat are in turn implicated in insulin resistance (IR) and dyslipidaemia [10], and associated with increased risk of metabolic syndrome, T2D and cardiovascular disease (CVD), independent of BMI [11–13].

Despite the important role that it may play, current assessment and accurate quantification of ectopic fat relies on either advanced imaging techniques or histologic examination of biopsied tissue, which are both time consuming and expensive, or invasive. Circulating biomarkers for early detection of visceral adiposity and ectopic fat deposition in key organs of liver and pancreas prior to the onset of metabolic disease are lacking. Notably, the critical importance of identifying such biomarkers has recently been highlighted by Neeland and colleagues in a position statement from the International Atherosclerosis Society and International Chair on Cardiometabolic Risk Working Group on Visceral Obesity [14].

With advanced metabolomics techniques, comprehensive measurements of plasma small molecules in combination with machine learning approaches may allow identification of novel biomarkers to estimate VAT and organ fat content from a single fasting blood sample. These markers may also reflect perturbed metabolism and point to underpinning mechanisms driving development of poor metabolic health. Systemic metabolomic profiling of non-alcoholic fatty liver disease (NAFLD) has identified candidate metabolite markers such as taurocholate, glutamyl dipeptides, mannose and lactate, carnitine and several acylcarnitines, FFA, lysophosphatidylcholine, glycerolipids (GL) as markers of NAFLD progression [15]. However, most biomarkers were identified in the context of diagnosed NAFLD cases, whereas biomarkers for early detection of asymptomatic liver fat deposition remain to be determined. Importantly, no circulating biomarkers of pancreas fat have yet been identified. To date only the targeted metabolomics study by Jaghutriz et al., have reported this data, in a study which was unable to identify plasma metabolites that characterised high vs. low pancreatic fat, in a group of prediabetic European Caucasians with impaired glucose tolerance [16]. More studies are required to determine whether markers of pancreatic fat deposition are detectable in circulation.

Our current cross-sectional study explored the relationship between plasma metabolome and fat deposition in the pancreas, liver and the ratio of visceral and subcutaneous abdominal adipose tissue (VAT/SAT ratio), assessed by magnetic resonance imaging (MRI) and spectroscopy (MRS), in Caucasian and Chinese participants enroled in the TOFI Asia study. The VAT/SAT ratio was of interest as it is an estimate of the relative body fat distribution, and that VAT is recognised as a more detrimental fat depot whereas SAT is generally considered neutral or even protective. The VAT/SAT ratio has been shown to be a key correlate of cardiovascular risk and events [17–19]. The goals of this study were firstly to identify candidate metabolite markers that predict pancreas, liver and VAT/SAT ratio, and elucidate the associated metabolic changes; and secondly to compare the predictive performance of these metabolite markers with a range of clinical measurements associated with each fat depot identified from the present cohort.

## Methods

### Study participants and protocol

This investigation is part of the cross-sectional TOFI_Asia study described elsewhere and the demographic and biochemical characterisation of the cohort is provided in Table S1 [20]. Female participants aged 20–70 years, BMI 20–45 kg/m^2^, fasting plasma glucose (FPG) ≤ 6.9 mmol/L,who self-reported both parents of same ethnicity (European Caucasian or Asian Chinese) were eligible. Exclusions were significant weight change (>10%) in prior 3 months, bariatric surgery, glucose-related medications, current/prior history of disease including T2D, pregnancy, breastfeeding. Sixty eight female participants (34 Chinese, 34 Caucasian) were enroled in the study. Fasting venous blood samples were collected in clinic and stored at −80 °C for later batch analyses. Total body fat (TBF) was determined by dual-energy X-ray absorptiometry (DXA) (iDXA, GE Healthcare, WI, USA) at the Body Composition Laboratory, University of Auckland. Magnetic resonance imaging (MRI) for pancreas and spectroscopy (MRS) for liver was conducted fasted within 1 week of clinic visit using a 3 T Magnetom Skyra Siemens scanner, VE 11 A (Erlangen, Germany) at the Centre for Advanced MRI (CAMRI), University of Auckland.

### Anthropometric and clinical measurements

Height, weight, waist and hip circumferences, systolic (SBP) and diastolic (DBP) blood pressure were recorded at clinic. Fasting plasma glucose was analysed by hexokinase method, HbA_1c_ by capillary electrophoresis (Cap2FP, IDF, France), liver function tests and lipid profile were analysed using standard clinical methods. Glucoregulatory peptides (insulin, C-peptide, glucagon, amylin, gastric inhibitory peptide (GIP), total glucagon-like peptide-1 (GLP-1)) were analysed using MILLIPLEX®MAP Human Metabolic Hormone Magnetic Bead Panel (Merck, HE, Germany) from BD P800 vautainers

### Assessment and analysis of body composition for visceral and organ fat

TBF and total body lean (TBL, fat-free soft tissue) mass were obtained from full body DXA scan, measured supine. TBF was expressed as % of total soft tissue mass:

%TBF = TBF mass*100/(TBL mass + TBF mass)

Fat content of abdomen (VAT, SAT), pancreas and liver were measured using MRI and MRS [21]. Briefly, the abdominal cavity was scanned in the sagittal direction from diaphragm to pelvis. A 2-point Dixon imaging technique was used for fat-water separation. Three blocks of forty 5-mm axial slices were acquired during an 11-s breath-hold. Pancreas was located, and fourteen axial images of 5-mm thickness acquired. For the MRS scan of liver, a 2*2*2 cm^3^ voxel was placed in the right lobe avoiding blood vessels and biliary tree; spectra were obtained in transverse, coronal and sagittal planes ± water suppression. Fat fraction (FF) map corrected for noise bias at L4-L5 intervertebral disc space was constructed using custom Matlab R2017a software (The Mathworks, Inc., Massachusetts, US), and abdominal adipose tissue from FF map segmented into VAT and SAT compartments (Image J [22]), and VAT/SAT ratio calculated. Pancreatic fat was estimated using the MR-opsy method as mean of 2 candidate pancreas FF maps, with 3 regions of interest (ROIs) head, body, tail [23]. For MRS, area under the curve (AUC) of water and fat peaks from spectra without water suppression were obtained (SIVIC software [24]), and liver fat expressed as % calculated vol/vol of fat and water. Pancreas FF maps from 3 Caucasians contained artefact, and MRS from 1 Caucasian could not be analysed due to inverse spectral signal; hence 65 pancreas, 67 liver fat, and 68 VAT/SAT ratios were analysed.

### Metabolomics analysis and data processing

Metabolite extraction, data acquisition and processing of samples from TOFI_Asia cohort have been previously described [25]. The processed metabolomic profile from samples in this current MRI cohort were isolated to construct a new dataset for statistical analyses. Briefly, metabolites were extracted using a bi-phasic approach and the aqueous and organic phases were analysed separately by two LC–MS platforms using a method published elsewhere [26]. Raw datafiles were converted to mzXML format with the ProteoWizard tool MSconvert (v 3.0.1818 [27]). Data preprocessing, cleaning, normalisation (by LOESS algorithm in the W4M Galaxy environment [28]), feature filtering (% coefficient of variation <30 in QC) and metabolites annotation were carried out. Full details are provided in SI.

### Data analysis

The statistical workflow is summarised in Fig. S1 in the SI. Biological outliers were removed based on partial least squares (PLS) residual analysis, and normality of response residuals re-assessed with the Kurtosis test (R v3.5.1) [29], details available in SI (Table S2 and Fig. S2). Multivariate methods with Unbiased Variable selection in R (MUVR) [30] were performed on the full metabolome (polar metabolites + lipids) to select important variables associated with pancreatic fat, liver fat, or VAT/SAT (continuous Y variable) in a PLS regression model (R v3.5.1). To ensure the model was not over-fitted before or after variable selection, 100 permutation tests were run on each PLS model with repeated-double cross-validation (PLS-rdCV) built on either full metabolome or MUVR-selected variables (R v3.5.1). Variables selected by MUVR were annotated and redundant chemometric features (e.g., isotopes, multiple adducts) representing the same metabolites were removed to constitute the post-selection data-matrix. Performances of PLS models built on full metabolome vs MUVR post-selection for each fat depot were compared, and β-coefficients of variables obtained (SIMCA 16, Umeå, Sweden). An optimal number of components were chosen to minimise root the mean square error of cross-validation (RMSEcv). The prediction accuracy was estimated by Pearson’s correlation coefficients (r) calculated between predicted vs measured Y values. To evaluate the predictive power of the set of metabolite markers and for comparison with potential clinical markers (including anthropometric parameters), sigfinicantly associated clinical markers with each fat depot were identified using linear regression (adjusting for ethnicity) and then combined to construct a panel for comparison of predictive power. Performance of PLS models built on (a) the panel of clinical markers, (b) the panel of metabolite markers, and (c) the panel of combined clinical and metabolite markers, were assessed and compared (SIMCA 16).To understand how individual metabolite related to each of pancreas, liver and VAT/SAT, linear regression with multiple testing correction (Benjamini Hochberg procedure (BH) [31]) was applied. The association between each metabolite and fat depot was corrected for ethnicity (model (M)1), then further for total adiposity-related parameters including BMI and %TBF (M2). For pancreatic and liver fat, the model was further adjusted for VAT/SAT (M3). Lastly, models for all three fat depots were further adjusted for age (M4).

## Results

### Identifying candidate markers of visceral and organ fat with a multivariate statistical approach

After metabolite selection by MUVR and removal of redundant features, 56 (91% identified), 64 (95% identified), and 31 (100% identified) variables were associated with pancreatic fat, liver fat, and VAT/SAT respectively. Comparison of model performances before and after variable selection are summarised in Table 1. In all 3 models, variable selection improved goodness of model fit (R2Y) and predictivity (Q2) whilst reducing the number of variables in the model. The fitted Y values using the selected variables were better correlated with the measured Y values (correlation coefficient r) than using the full metabolome. The number of components in each PLS model was selected such that it achieved minimum prediction error, determined by assessing model prediction accuracy and average error (Q2 and RMSEcv) (Fig. S3). Overfitting of models was avoided by assessing model performances with rdCV as an alternative validation scheme with 100 permutations (Table S3).

**Table 1:** Comparison of model performances of PLS with sevenfold cross-validation between full metabolome vs post-selection.

		Full metabolome					Post-selection					
	nVar	nComp	R2Y	Q2	P_CV-ANOVA_	*r*	nVar	nComp	R2Y	Q2	P_CV-ANOVA_	*r*
Pancreatic fat	910	1	0.39	0.25	2.43E–04	0.63	56	3	0.81	0.69	2.12E–12	0.90
Liver fat	910	1	0.52	0.33	6.86E–06	0.72	64	3	0.80	0.66	6.11E–12	0.89
VAT/SAT	910	1	0.47	0.30	1.32E–05	0.69	31	2	0.70	0.62	1.88E–12	0.83

nVar: number of variables; nComp: number of components; R2Y: goodness of model fit; Q2: predictivity; Pcv-anova: statistical significance of the PLS model; r: Pearson correlation coefficient.

**Fig. 1:**
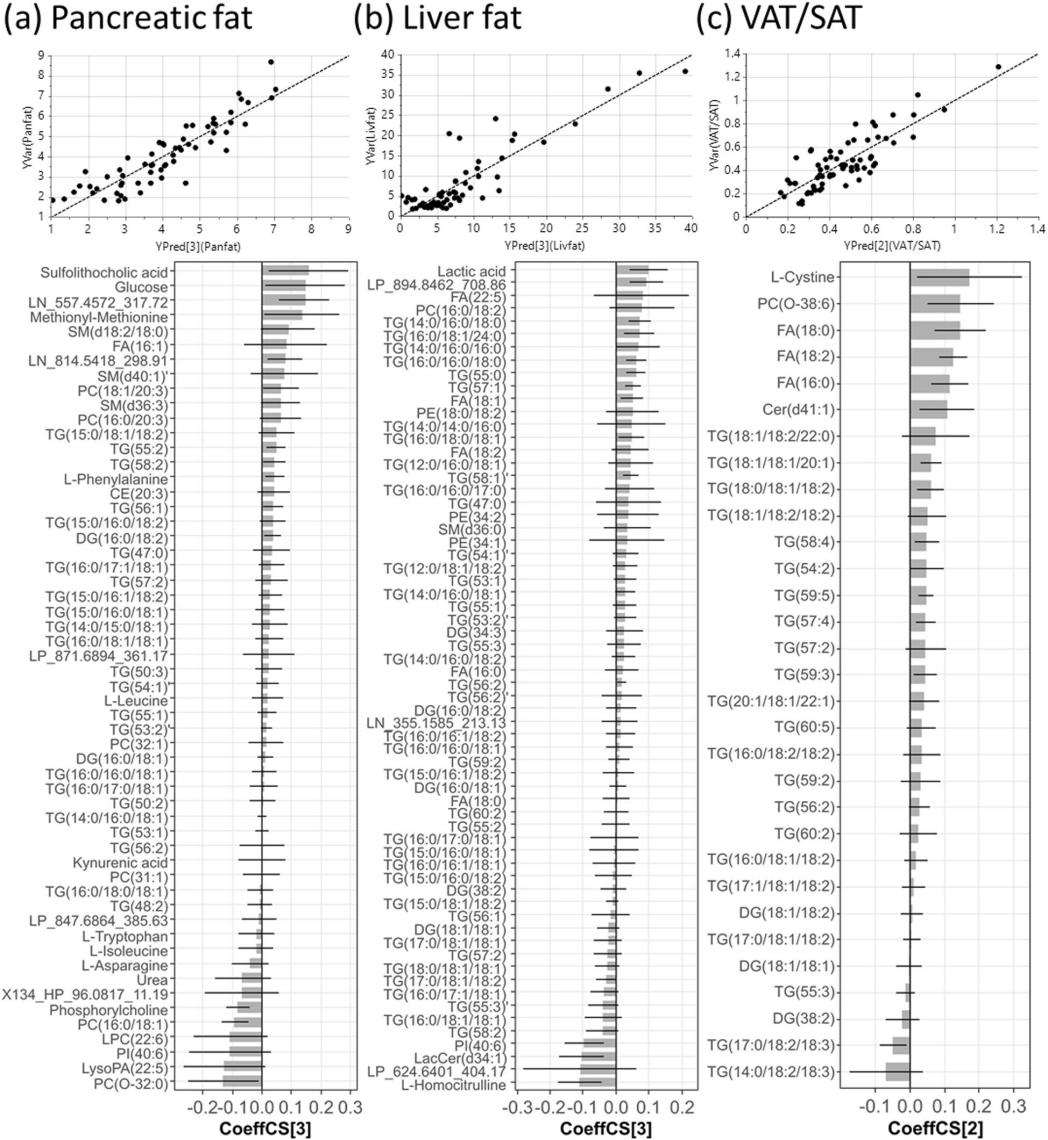
Partial least squares (PLS) regression analysis of ectopic fat deposition. Scatter plots of measured vs PLS-estimated value of (**a**) pancreatic fat, (**b**) liver fat and (**c**) VAT/SAT ratio, and bar plots showing the coeffCS (centred and scaled coefficient) with error bars indicating cross-validation confidence interval (95% CI) of variables in each corresponding PLS model used for estimation. The numbers in the bracket beside YPred and CoeffCS denotes the number of components in the PLS model.

Pancreatic fat was associated with sulfolithocholic acid, cholesteryl ester (CE(20:3)), fatty acid (FA(16:1)), LC-MS measured glucose, urea, phosphorylcholine, kynurenic acid, 5 amino acids (AA) and lipid species encompassing 26 glycerolipids (GL), 9 glycerophospholipids (GP) and 3 sphingolipids (SP) (Fig. 1a). These metabolites jointly explained 81% of the variance of pancreatic fat in a 3 component-PLS model and estimated levels of pancreatic fat with a high correlation with measured levels (*r* = 0.90). Liver fat was associated with homocitrulline, lactate, lactosylceramide (LacCer(d34:1)), dihydrosphingomyelin (dhSM(d36:0)), 5 FAs, 47 GLs and 5 GPs (Fig. 1b). These metabolites jointly explained 80% of the variance of liver fat in a 3 component-PLS model and estimated levels of liver fat with a high correlation with measured levels (*r* = 0.89). Notably, the TG species associated with liver fat were highly saturated (all containing ≤3 unsaturated bonds); among the 27 MS2-annotated TGs, 75% contained at least one C16:0 palmitic acid (PA), while 63% contained at least one C18:1 oleic acid (OA). VAT/SAT was associated with L-cystine, ceramide (Cer(d41:1)), ether linked phosphatidylcholine (PC(O-38:6)), 3 FAs and 25 GLs (Fig. 1c). These metabolites jointly explained 70% of the variance of VAT/SAT in a 2 component-PLS model and estimated levels of VAT/SAT with a high correlation with measured levels (*r* = 0.83). Among the 11 MS2-annotated TGs associated with VAT/SAT, 82% contained at least one C18:2 linoleic acid (LA), and 73% contained at least one C18:1 OA.

### Estimating visceral and organ fat deposition using clinical and candidate metabolite markers

**Fig. 2:**
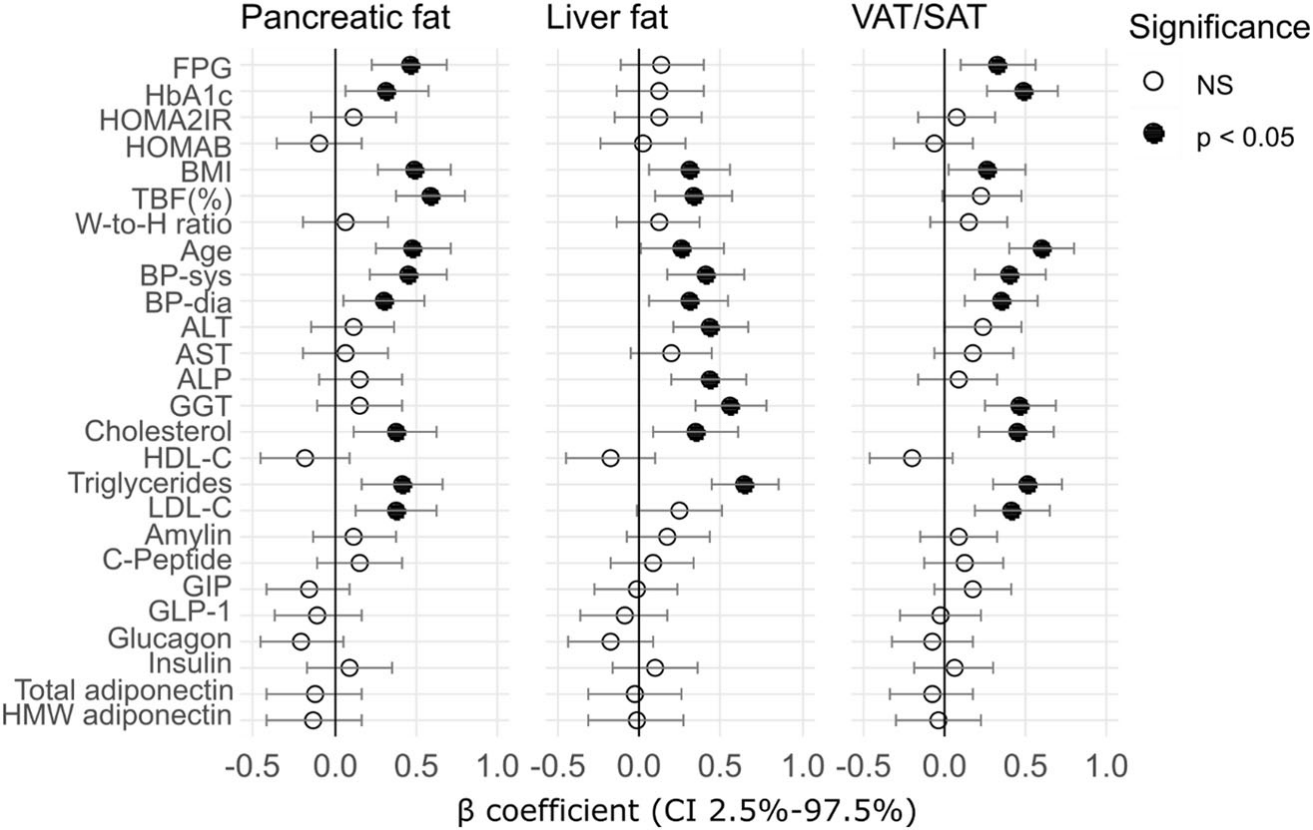
Association of clinical measurements with ectopic fat deposition. Adjusted β-coefficient with 95% CI of each individual clinical variable regressed on level of (**a**) pancreatic fat, (**b**) liver fat and (**c**) VAT/SAT ratio in linear regression models adjusting for ethnicity. Significantly associated clinical variables (*p* < 0.05) are displayed in solid filled circle.

Pancreatic fat was associated with FPG, HbA_1c_, BMI, %TBF, age, SBP, DBP, total cholesterol (TC), triglyceride (TG) and LDL-C (Fig. 2a), which jointly produced a PLS model with R2Y = 0.51, Q2 = 0.46 (Table 2). Liver fat was associated with BMI, % TBF, age, SBP, DBP, ALT, ALP, GGT, TC and TG (Fig. 2b), yielding a PLS model with R2Y = 0.48, Q2 = 0.4 (Table 2). VAT/SAT was associated with FPG, HbA_1c_, BMI, age, SBP, DBP, GGT, TC, TG and LDL-C (Fig. 2c), producing a PLS model with R2Y = 0.56, Q2 = 0.44 (Table 2). Metabolite markers explained the presence of pancreatic fat (R2Y = 0.81, Q2 = 0.69), liver fat (RY2 = 0.8, Q2 = 0.66) and VAT/SAT (R2Y = 0.7, Q2 = 0.62) better than clinical markers; while combining clinical markers and metabolite markers yielded a similar model performance compared to the use of metabolite markers alone (Table 2).

**Table 2:** Comparison of model performances of PLS with sevenfold cross-validation among models built on metabolites markers (M), clinical markers (C) or combination of metabolite and clinical markers (C + M).

Dataset	Biomarker panel	nComp	R2Y	Q2	P_CV-ANOVA_	RMSEE	RMSEcv	*r*
Pancreatic fat	M	3	0.81	0.69	2.12E–12	0.72	0.89	0.90
	C	1	0.51	0.46	1.15E–08	1.14	1.18	0.71
	C + M	3	0.81	0.67	1.12E–11	0.72	0.92	0.90
Liver fat	M	3	0.80	0.66	6.11E–12	3.84	4.65	0.89
	C	1	0.48	0.40	3.51E–07	5.98	6.37	0.70
	C + M	2	0.74	0.63	5.49E–12	4.25	4.94	0.86
VAT/SAT	M	2	0.70	0.62	1.88E–12	0.13	0.14	0.83
	C	2	0.56	0.44	1.42E–07	0.16	0.17	0.74
	C + M	2	0.74	0.67	3.98E-14	0.12	0.13	0.86

nComp: number of components; RMSEE: roo mean square error of estimation; RMSEcv: root mean square error of cross-validation; r: Pearson correlation coefficient.

### Characterising the association of individual metabolite markers with visceral and organ fat

**Fig. 3:**
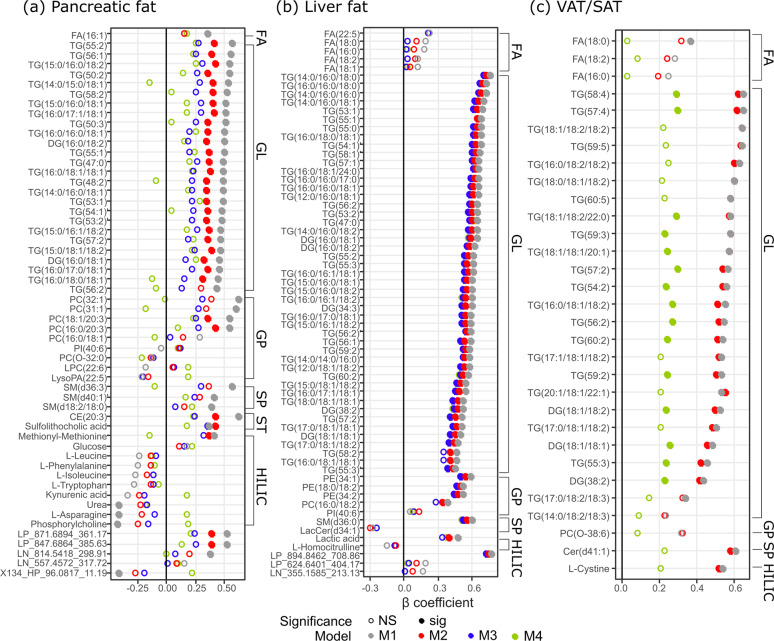
Association of individual metabolite marker with ectopic fat deposition. Association of individual metabolite markers with (**a**) pancreatic fat, (**b**) liver fat, (**c**) VAT/SAT ratio assessed by linear regression, with adjustment for ethnicity (M1), ethnicity + BMI + TBF(%) (M2); pancreatic fat and liver fat were further adjusted for VAT/SAT (M3); and additionally for age (M4). Significance tests were after multiple testing correction (*p* < 0.05). For the (**b**) liver fat age-adjusted model (M4, green) did not alter the β-coefficient therefore dots were mostly overlapped with M3; details of the β-coefficient values can be found in Table S5. Markers were categorised by classes and within each class, ordered by β-coefficient in M1 from the highest to the lowest. FA fatty acyls, GL glycerolipids, GP glycerophospholipids, SP sphingolipids, ST sterol lipids, HILIC HILIC-measured polar metabolites.

Of the 56 metabolites associated with pancreatic fat, 44 were independent of ethnicity (BH-corrected *p* < 0.05). Further adjustment for total adiposity yielded a list of lipid species of PC, DG and TG, CE(20:3), methionyl-methionine and sulfolithocholic acid as significantly associated metabolites (BH-corrected *p* < 0.05); only the bile acid sulfolithocholic acid remained significantly associated after further adjustment for VAT/SAT (M3). No metabolite remained significantly associated after futher adjustment for age (M4) (Fig. 3a and Table S4). Among the 64 metabolites associated with liver fat, 54 were independent of ethnicity and total adiposity (BH-corrected *p* < 0.05). Only lactic acid, PC(16:0/18:2), TG(16:0/18:1/18:1) and TG(58:2) became non-significant after further adjustment for VAT/SAT; none of these associations was influenced by futher adjustment for age (Fig. 3b and Table S5). Among the 31 metabolite markers for VAT/SAT, 27 were independent of ethnicity and further adjustment for total adiposity did not alter these associations (BH-corrected *p* < 0.05) except FA(18:0) and TG(53:5), which became non-significant. Only 15 metabolites including 3 DGs and 12 TGs remained significantly associated with VAT/SAT when age was further included in the regression model (Fig. 3c and Table S6).

## Discussion

In this study, we identified plasma metabolites associated with visceral and ectopic fat deposition in a cohort of European Caucasian and Asian Chinese females, in alignment with recent international position statements for the need for identification of such biomarkers in clinical practice [14]. With a robust metabolite selection technique and PLS modelling approach, novel metabolite markers that jointly explain over 70% variance (i.e., R2Y) in pancreatic fat, liver fat and VAT/SAT ratio have been identified. The estimated levels of these fat depots by metabolite markers were highly correlated with the measured values (*r* > 0.8). We also assessed the associations of a range of commonly used clinical and anthropometric measurements with these fat depots. Several traditional CVD risk factors, including age, BP (SBP, DBP) and dyslipidaemia (TC and TG), were common correlates for all three fat depots. Despite this, these CVD risk factors alone with other significant clinical correlates for each fat depot, only moderately captured levels of ectopic fat (R2Y at around 0.5) and did not add to the metabolite prediction of ectopic fat. Our study highlighted the value of biomarker exploration using an untargeted metabolomics approach. These markers hold promise for developing new means to predict levels of ectopic fat which is otherwise expensive and time-consuming to obtain, but also provide insight into metabolic alterations that might be linked to dietary pattern and disease development thus generating hypothesis for future intervention or mechanistic studies. Furthermore, these metabolite markers can be a fast and cheap substitute for the MRI/MRS-assessed ectopic fat to be included for an improved prediction of cardiometabolic disease outcomes, which will need to be evaluated in future prospective studies.

To our best knowledge, this is the first study reporting novel metabolite markers predictive of pancreatic fat content, among which the bile acid (BA), sulfolithocholic acid, remained significantly associated with pancreatic fat independent of total and visceral adiposity, though this association became non-significant after further adjustment for age. Age is a factor impacts considerably on regional fat distribution [32], and finding from our relatively small cohort suggested the observed association between BA and pancreatic fat deposition is likely to be confounded by the effect of age. To elucidate the potential value of sulfolithocholic acid as a marker for predicting pancreatic fat, the interrelationship among age, sulfolithocholic acid and pancreatic fat needs to be clarified using another larger study cohort. Nonetheless, altered BA metabolism and signalling are implicated in both T2D development and bariatric surgery-induced metabolic improvements [33, 34]. Sulfolithocholic acid is the sulphated product of lithocholic acid (LCA), a secondary BA produced by microbiota [35]. Levels of secondary BAs were observed to increase after bariatric sugery and associated with improved glycaemic control [36, 37]. Since both adipocytes and pancreatic cells expressed BA receptors [33], the profound association of sulfolithocholic acid with pancreatic fat observed in the present study sheds new light on potentially novel mechanisms of fatty infiltration modulated by the enterohepatic circulation and BA metabolism. A future targeted metabolomics study is warranted to confirm this association as well as to explore the relationship between other BAs with pancreatic fat deposition.

Other markers predictive of pancreatic fat identified in the present study included a number of metabolites previously reported as markers of obesity (palmitoleic acid, monounsaturated PCs and SMs, asparagine, phosphorylcholine and urea) [38–43]. Concordantly, depot-specific investigation of these markers in our current study indicates that their associations with pancreatic fat were, indeed, largely due to increased total adiposity. Other markers including CE(20:3), C20:3-containing PCs, DGs, TGs and methionyl-methionine were independent of BMI and %TBF, but explained by increased VAT/SAT. Since both increased total adiposity and visceral adiposity are upstream factors predisposing individuals to an increased risk of ectopic fat deposition, our results suggest that these markers for pancreatic fat mainly reflect increased total and visceral adiposity.

We observed a TG signature characterised by low double bonds (≤3) and mainly lower carbon number (≤54 C) to be associated with liver fat content. TG species with low double bonds and carbon number were found to be markerdly increased in NAFLD patients and have the strongest predictive value in the classification of incident CVD, whilst monounsaturated TG was a significant predictor of non-alcoholic steatohepatitis (NASH) [44–46]. Our findings suggested that such a CVD risk- and NAFLD-related TG signature to some degree captured an elevated liver fat content and is already detectable even before NAFLD diagnosis, again highlighting the potential of metabolomics to facilitate risk screening.

Interestingly, several saturated TG species such as TG(44:0), TG(46:0), TG(48:0) and TG(50:0) are markers exclusively for liver fat but not visceral or pancreatic fat. These markers may reflect consumption of a high saturated fat diet [47]. Many liver fat-associated TG species contained PA and/or OA, both are hallmarks of *de novo* lipogenesis (DNL). Higher rate of DNL, fractional contribution to VLDL-FA and VLDL-TG from DNL were previously observed in patients with higher liver fat contents, and DNL rate positively correlated with the amount of intrahepatic TG [48]. In agreement, our data clearly indicated that accumulation of liver fat can be manifested by an elevation of a consortium of TG species in the circulation that likely originated from DNL, independent of the total and visceral adiposity. Our results support a tight link between increased DNL and development of fatty liver, as well as potential beneficial effects of dietary intervention targeting lipogenesis, possibly including restricted mono/disaccharide carbohydrate or elimination of fructose-containing diets alongside restricted fat diets [49, 50].

Liver fat was also associated with dhSM(d36:0) and 3 PE species (PE(34:1), PE(34:2) and PE(36:2)) independent of total adiposity and visceral adiposity. Our findings are in line with a previous study showing increased concentration of circulating PE relative to progression of fatty liver disease [51]. On the other hand, dhSM has been associated with obesity and dysglycaemia [52]. Our finding of a positive correlation of dhSM(d36:0) with liver fat could be due to an increased substrate availability leading to increased synthesis of this lipid species, supporting the aforementioned increased DNL associated with excess liver fat deposition.

Two other markers, PC(34:2) and lactate, were also strongly associated with and predictive of, but not site-specific to, liver fat content. This is not unexpected as these metabolites are localised in many tissues, abundant in plasma and sensitive to several conditions and diseases. Increased plasma lactate has been associated with impaired oxidative capacity, obesity, IR and T2D, and its level progressively decreased in response to weight loss [53–56]. PC(34:2) has been identified as a marker for metabolic syndrome [57]. It is also associated with vascular complications in NAFLD patients [58]. Herein we provided evidence for an association of these markers with increased liver fat and in conjunction with other metabolites markers, predictive of liver fat content.

VAT/SAT is associated with a number of GLs enriched in LA. LA is an n-6 FA proposed to be obesogenic and may contribute to a chronic inflammatory state due to competition with the n-3 FA alpha-linolenic acid (ALA) for Δ-6 desaturase [59]. Whilst n-6 FAs are precursors for pro-inflammatory mediators, the n-3 FAs products have lower inflammatory or even anti-inflammatory properties. Interestingly, 2 ALA-containing TG species were observed to be the strongest negative estimators for the level of VAT/SAT in the multivariate PLS model, as opposed to several LA-containing TGs that are positively associated. Furthermore, LA along with 2 saturated FAs appeared to be strong estimators for the level of VAT/SAT in the PLS model as their β-coefficients were high (ranked 3rd–5th). Since the plasma TG signature may also reflect long-term dietary patterns, such associations suggest a possible link between long-term consumption of LA-rich diets and increased visceral adiposity.

Plasma levels of cystine taken as an indicator of oxidative stress, has been previously shown to be associated with android (visceral) fat at baseline in a cohort of patients with a cardiometabolic disorders [60]. In agreement, our result reported L-cystine as a significant predictor for and strongly correlated with VAT/SAT independent of total adiposity. However, this association became non-significant after age adjustment. Since age is also tightly associated with VAT/SAT ratio as shown by the present study, whether plasma L-cystine could effectively capture VAT/SAT will need to be investigated in a large, age-controlled cohort. Another factor that may affect plasma level of L-cystine but not taken into account by the present study is dietary methionine consumption [61, 62]. It would be necessary to investigate through a randomised controlled trial (RCT) whether a methionine-restricted diet can reduce circulating cystine level and simultaneously reduce visceral adiposity and improve metabolic health.

A strength of our present study is the use of a robust variable selection technique MUVR to select candidate markers, which has effectively reduced the risk of overfitting, biased selection and false positive discoveries [30]. Secondly, this study was conducted on a cohort free of severe metabolic diseases or medications, thus maximally eliminating potential confounding effects of metabolic diseases on the blood metabolome, allowing the identification of candidate markers for early detection of increased risk of poor metabolic health prior to disease onset. With both DXA and MRI/MRS data available we were able to adjust the association for TBF, enhancing the understanding of the site-specificity of these markers. Limitations of this study include the relatively small cohort size, a non-controlled diet study design, and the lack of an external validation cohort, which must be addressed in future studies. The lack of dietary data and that participants were not on the same diet before sample collection made it impossible to account for the effect of diet on the observed associations. Last, although the predicted Y value can be theoretically calculated using solely the β-coefficients provided in the PLS model, these β-coefficients are not transferable across platforms and laboratories. Thus, translation of these candidate markers to potential clinical biomarkers will require accurate quantification of absolute concentration by other techniques e.g., targeted LC-MS/MS.

In conclusion, we have identified metabolites predictive of ectopic fat deposition (pancreas and liver fat, and VAT/SAT ratio) and shown these candidate metabolite markers to outperform the use of anthropometric and clinical measurements, including several CVD risk factors. Importantly, sulfolithocholic acid is a novel marker for pancreatic fat which requires validation in the future. Other markers are consistent with findings from previous metabolomics studies in the context of obesity, fatty liver diseases, T2D and CVD. Noteworthy, our cohort is devoid of cardiometabolic diseases therefore these markers held promise for developing alternative approach for detection of increased ectopic fat deposition and risk screnning prior to disease onset. Moreover, the metabolite markers provide evidence at the molecular level potentially linking ectopic fat to dietary intake, generating hypotheses for future investigation. These markers may also provide an alternative means to measure the effectiveness of dietary interventions. Whether these markers add value to the prediction of cardiovascular risk will require further study and validation in separate cohorts.

## Supplementary information

Supplementary information

Supplementary table 1

Supplementary table 2

Supplementary table 3

Supplementary table 4

Supplementary table 5

Supplementary table 6
