# Supplementary material for: Untargeted metabolomics reveals plasma metabolites predictive of ectopic fat in pancreas and liver as assessed by magnetic resonance imaging: the TOFI_Asia study
Source: Int J Obes (Lond). 2021 May 16;45(8):1844–54. doi: 10.1038/s41366-021-00854-x (PMC8310794; doi:10.1038/s41366-021-00854-x)
Supplement: Supplementary file 1 — Supplementary information [file 41366_2021_854_MOESM1_ESM.docx]

**Supplementary Information**

**Metabolomics profiling, data processing and metabolite annotation**

Metabolites were extracted using a bi-phasic approach by mixing 100 µL plasma with 800 µL pre-chilled (-20 °C) CHCl_3_:MeOH (50:50, v/v) followed by addition of 400 µL H_2_0. 200 µL of the upper aqueous layer and 200 µL of the lower organic layer were separately dried down and stored at -80 °C. Blank samples were prepared following the same protocol replacing plasma with H_2_0. Pooled QC samples were prepared by combining an aliquot of the upper or lower phase from every study sample and dispensed into 200-µl aliquots.

On the day of instrumental analysis, dried aqueous extracts were reconstituted in 200 µL acetonitrile:H_2_O (50:50, v/v). Reconstituted aqueous extracts were analysed using a Thermo LC–MS system consisting of an Accela 1250 quaternary UHPLC pump coupled to an Exactive Orbitrap mass spectrometry (Thermo Fisher Scientific, Waltham, MA, USA) with electrospray probe operated unheated at room temperature (20 °C). Polar metabolites were separated at 25 °C on a SeQuant® ZIC®-pHILIC 5 µm, 2.1 mm × 100 mm column (Merck, Germany) with solvent system A = 10 mM ammonium formate in water, B = 0.1% formic acid in acetonitrile. A gradient program was used at a flow rate of 250 µL/min: 3–3% A (0.0–1.0 min), 3–30% A (1.0–12.0 min), 30–90% A (12.0–14.5 min), 90% A was maintained for 3.5 min followed by re-equilibration with 3% A for 7 min. An injection volume of 2 µL was used. External mass calibration of the Orbitrap prior to sample analysis was performed by flow injection of the calibration mix solution according to the manufacturer’s instruction. High resolution data (resolution 25,000) were acquired by full scan from m/z 55 to 1100 with source voltage of 4000 V for ESI+ and −4000 V for ESI−, capillary temperature of 325 °C, and sheath, auxiliary, and sweep gas flow rates of 40, 10, and five arbitrary units, respectively.

Dried organic extracts were reconstituted in 200 µL modified Folch solution (CHCl_3_:MeOH:H_2_O, 66:33:1, v/v/v) containing pre-dissolved 0.01% 16:0 d_31_-18:1-PE phosphatidylethonolamine (PE) internal standard [0.01% (%w/v)]. Reconstituted organic extracts were analysed by Thermo LC–MS system consisted of an Accela 1250 quaternary UHPLC system coupled to Q Exactive hybrid quadrupole-Orbitrap mass spectrometer (Thermo Fisher Scientific, Waltham, MA, USA) with a heated electrospray ionisation source set to 370 °C. Lipids were separated on an Acquity CSH™ C18 column 1.7 µm, 2.1 mm × 100 mm (Waters, USA) maintained at 65 °C with mobile phases comprised acetonitrile/H2O (60:40) with 10 mM ammonium formate and 0.1% formic acid (A), and isopropanol/acetonitrile (90:10) with 10 mM ammonium formate and 0.1% formic acid (B). Analytes were eluted from the column with the following gradient program: 15–30% B (0.0–2.0 min), 30–48% B (2.0–2.5 min), 48–82% B (2.5–11.0 min), 82–99% B (11.0–11.5 min), 99% B was maintained for 3.5 min followed by re-equilibration with 15% B for 3 min at a flow rate of 600 µL/min. External mass calibration of the Orbitrap prior to sample analysis was performed by flow injection of the calibration mix solution according to the manufacturer’s instructions. High resolution data (resolution 70,000) was acquired by full scan from m/z 200–2000 with source voltage of 3500 V electrospray ionisation positive mode (ESI+) or −3600 V ESI negative mode (ESI−), capillary temperature of 275 °C, and sheath, auxiliary and sweep gas flow rates of 40, 10 and 5 arbitrary units, respectively. Data-dependent MS2 data were collected with a mass resolution set to 35,000 recording a mass range of m/z 200-2000 and maximum trap fill time of 250 ms (full scan mode) or 120 ms (MS2 scan mode). The isolation window of selected MS1 scans was ± 1.5 m/z with a normalised collision energy of 30 units.

Raw datafiles were converted to mzXML format with the ProteoWizard tool MSconvert (v 3.0.1818 [1]). Lipid data were pre-processed with XCMS (v3.0.2) in an R environment (v3.2.2) [2]. Polar metabolite data were pre-processed with the ADAP algorithm in mzMINE (v2.31 [3]) [4]. Data cleaning, normalisation (by LOESS algorithm in the W4M Galaxy environment [5]), and feature filtering (% coefficient of variation < 30 in QC) were also carried out to generate a data matrix for statistical analysis. Lipids were annotated using LipidSearch software v4.1.16 on MS^2^ datafiles (Thermo Fisher Scientific, USA). Polar metabolites were annotated using an in-house library based on authentic standards (AgResearch) analysed through HILIC LC–MS analysis under conditions identical to the current study. Unidentified features were searched against online databases including HMDB (http://www.hmdb.ca/), Metlin (http://metlin.scripps.edu/) and Lipid Maps (http://www.lipidmaps.org/) based on m/z with less than 10 ppm (for lipid features) or 15 ppm (for polar metabolite features) mass error.

**Determination and exclusion of biological outliers**

A partial least square (PLS) regression model was initially applied to examine the relationship between the level of each fat depot measured by MRI and the plasma metabolome (lipids + polar metabolic features) in the whole MRI cohort. In our preliminary exploration, a significant PLS model was only observed for VAT/SAT ratio (p < 0.05) (**Table S1**). Examination of residuals suggested the presence of biological outliers (i.e. the relationship between the level of fat depot and the metabolomic profile of these samples did not follow the general pattern of the rest of the cohort) (**Figure S2**). Exclusion of outliers improved the residual normality and goodness of model fit, suggesting the poor model performances, particularly of pancreatic fat and liver fat, were driven by these few outliers (**Table S1**). As such, for the subsequent analysis outliers were excluded (3 samples for pancreatic fat, 0% Caucasian; 5 samples for liver fat, 60% Caucasian; 1 sample for VAT/SAT, 100% Caucasian).

**Figure S1**

**
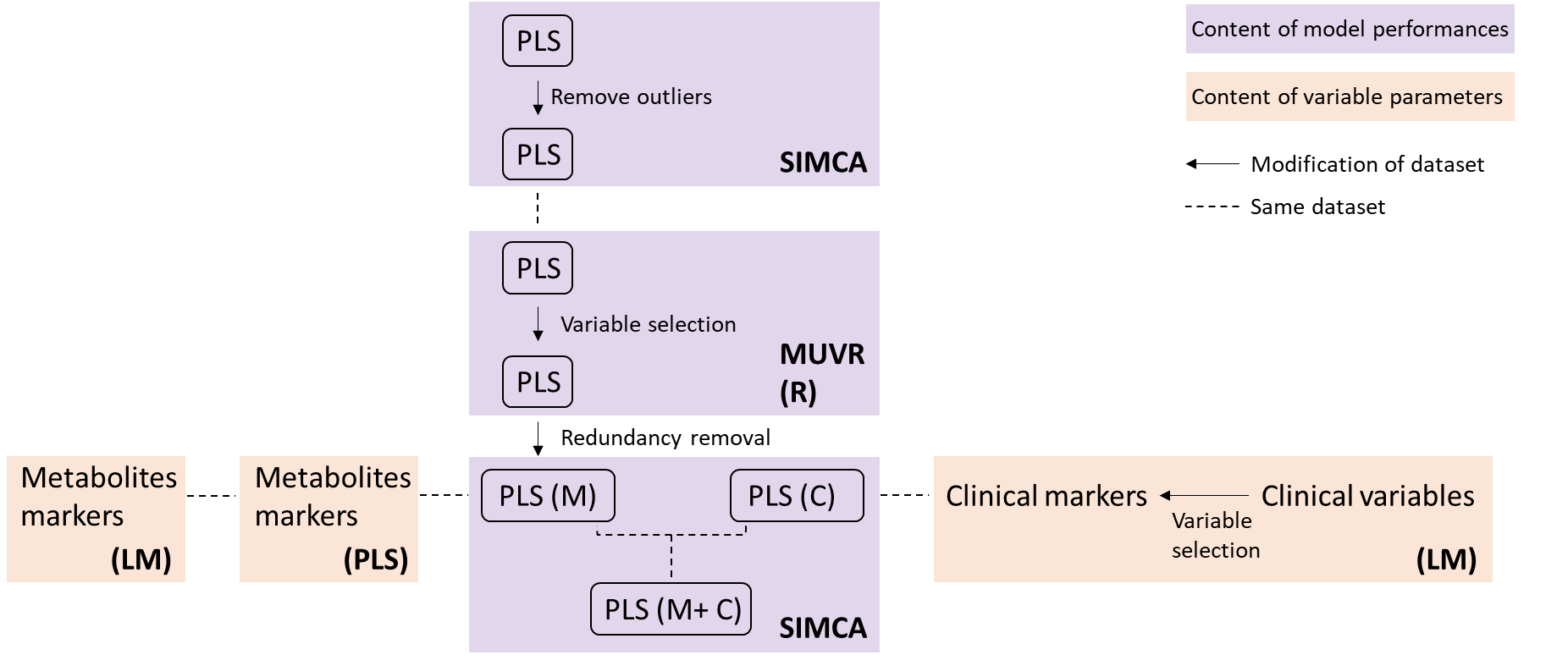
**

**Figure S1:** Workflow for data analysis in this study. SIMCA: SIMCA software v16; LM: linear regression; PLS: partial least squared regression; MUVR:

**Figure S2**

**
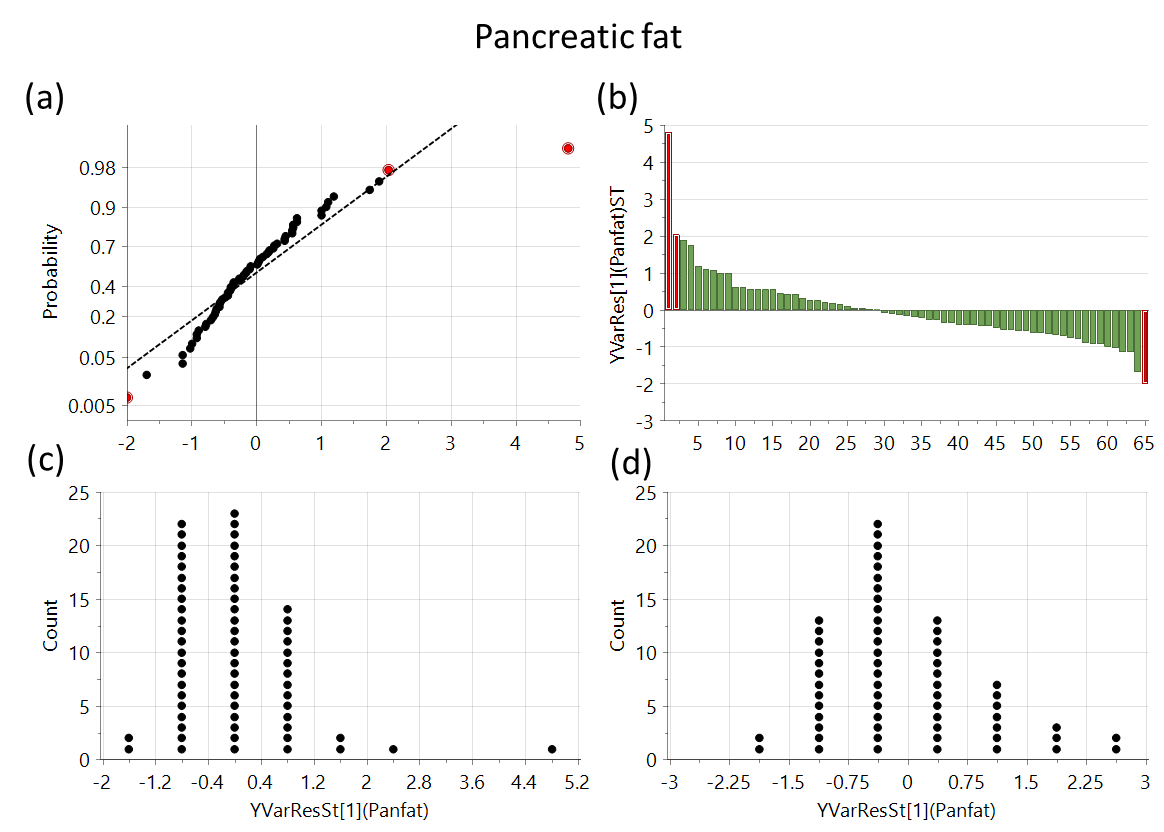
**

**
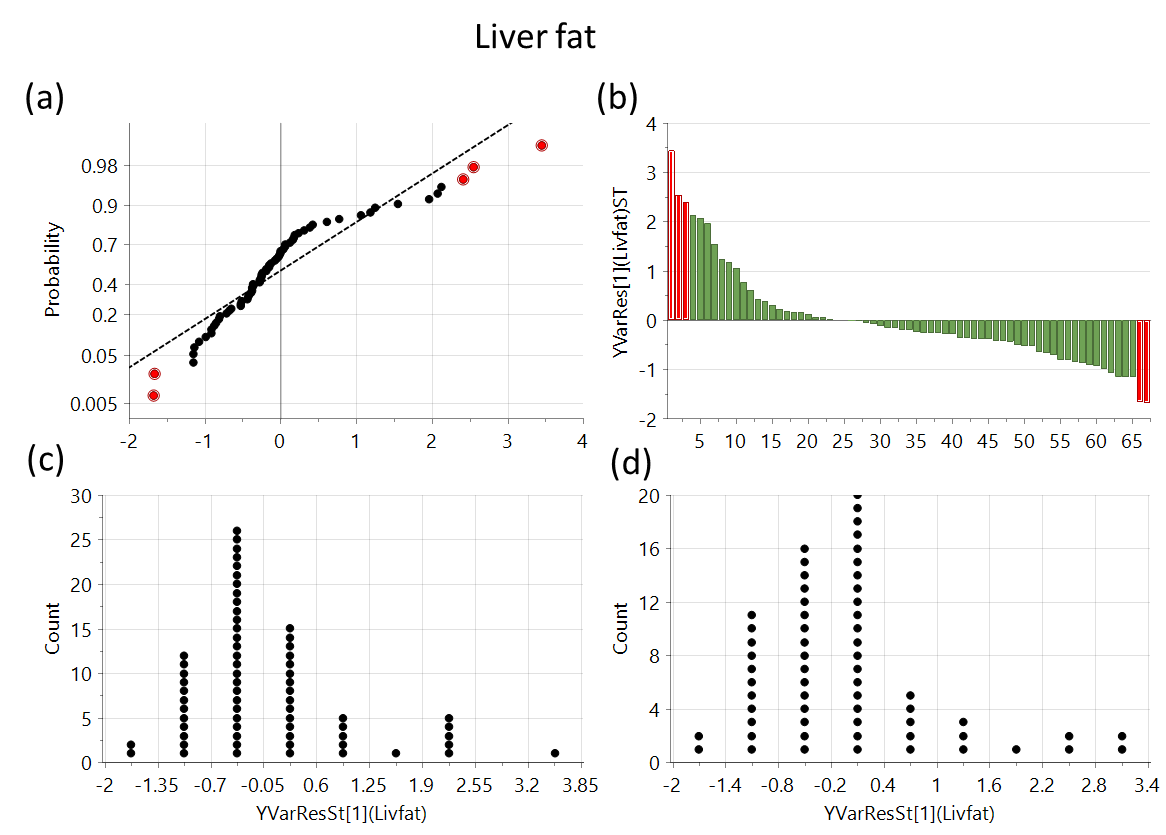
**

**
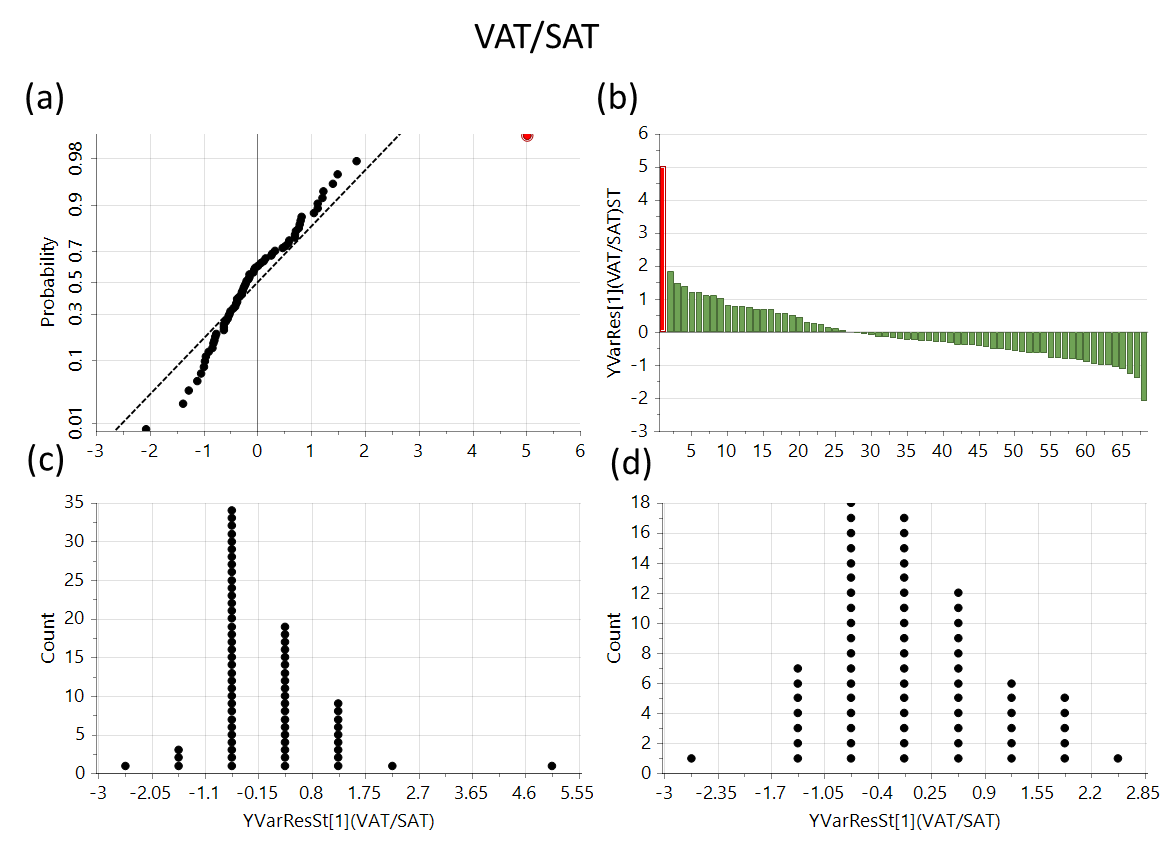
**

**Figure S2**: Y residual probability N-plot (a), bar plot showing Y residual expressed as unit of standard deviation (SD) (b), dot plot showing distribution of Y residual expressed as unit of SD before (c) and after (d) exclusion of outliers. The excluded outliers were highlighted in red in (a) and (b).

**Figure S3**


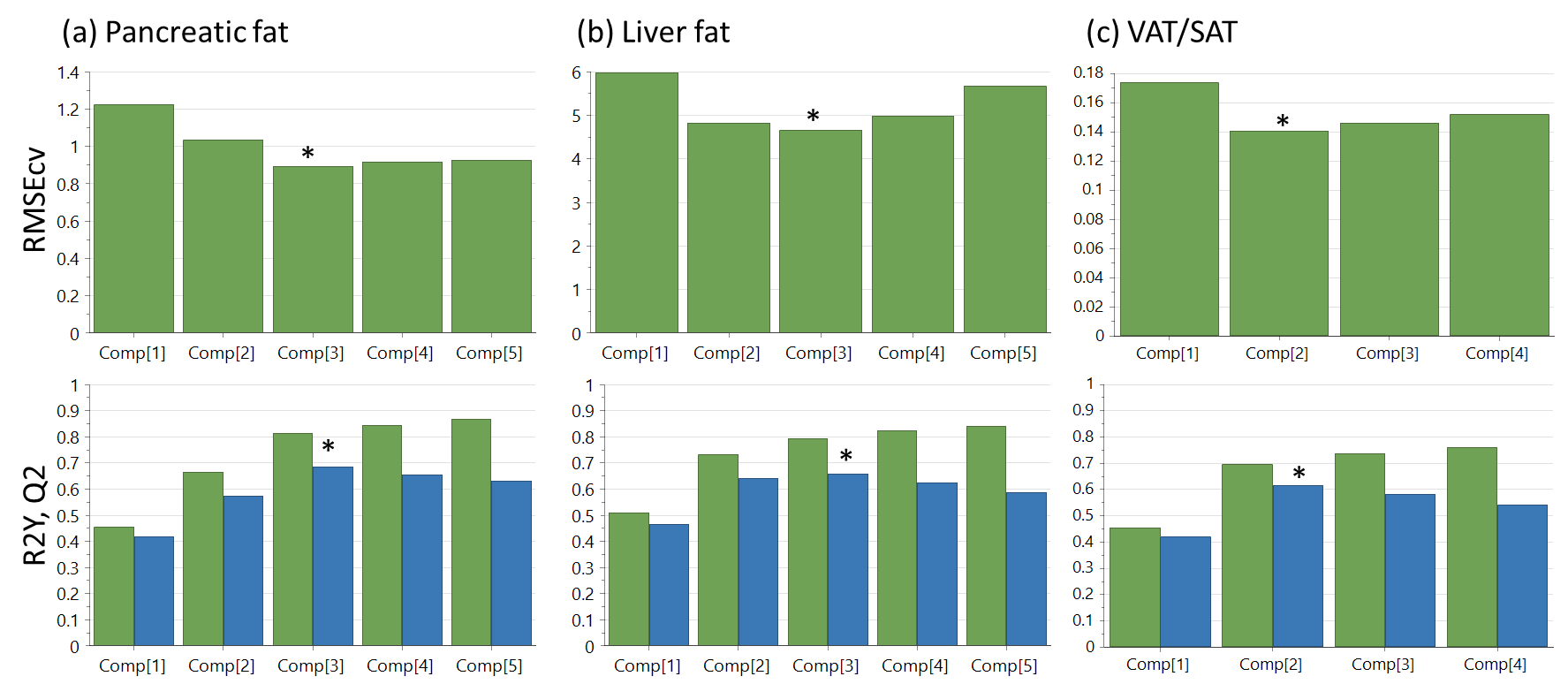


**Figure S3**: barplot showing RMSEcv (top) and model performance (bottom) of up to 5 components in (a) pancreatic fat, (b) liver fat, (c) VAT/SAT. Number of components producing the lowest RMSEcv and highest Q2 (blue) was chosen as optimum (marked as *).

Reference:

1. Chambers MC, Maclean B, Burke R, Amodei D, Ruderman DL, Neumann S, et al. A cross-platform toolkit for mass spectrometry and proteomics. Nat Biotechnol. 2012;30(10):918-20.

2. Smith CA, Want EJ, O'Maille G, Abagyan R, Siuzdak G. XCMS: processing mass spectrometry data for metabolite profiling using nonlinear peak alignment, matching, and identification. Anal Chem. 2006;78(3):779-87.

3. Katajamaa M, Miettinen J, Orešič M. MZmine: toolbox for processing and visualization of mass spectrometry based molecular profile data. Bioinformatics. 2006;22(5):634-6.

4. Myers OD, Sumner SJ, Li S, Barnes S, Du X. Detailed investigation and comparison of the XCMS and MZmine 2 chromatogram construction and chromatographic peak detection methods for preprocessing mass spectrometry metabolomics data. Anal Chem. 2017;89(17):8689-95.

5. Van Der Kloet FM, Bobeldijk I, Verheij ER, Jellema RH. Analytical error reduction using single point calibration for accurate and precise metabolomic phenotyping. J Proteome Res. 2009;8(11):5132-41.
